# Calcium enhances polyplex-mediated transfection efficiency of plasmid DNA in Jurkat cells

**DOI:** 10.1080/10717544.2020.1770371

**Published:** 2020-06-03

**Authors:** V. S. S. Abhinav Ayyadevara, Kyung-Ho Roh

**Affiliations:** a Biotechnology Science and Engineering, University of Alabama in Huntsville, Huntsville, AL, USA; b Department of Chemical and Materials Engineering, University of Alabama in Huntsville, Huntsville, AL, USA

**Keywords:** T-cells, Jurkat, poly(ethylene imine), calcium chloride, divalent cation, transfection, gene delivery, gene therapy, polyplex, lipoplex

## Abstract

Jurkat, an immortalized cell line derived from human leukemic T lymphocytes, has been employed as an excellent surrogate model of human primary T-cells for the advancement of T-cell biology and their applications in medicine. However, presumably due to its T-cell origin, Jurkat cells are very difficult to transfect. Thus, for the genetic modification of Jurkat cells, expensive and time-consuming viral vectors are normally required. Despite many previous efforts, non-viral vectors have not yet overcome the hurdles of low transfection efficiency and/or high toxicity in transfection of Jurkat cells. Here, we report that a simple addition of calcium ions (Ca^2+^) into culture media at optimal concentrations can enhance the efficiency of the polyplex-mediated transfection using poly(ethylene imine) (PEI) by up to 12-fold when compared to the polyplex-only control. We show that calcium enhances the association between polyplex and Jurkat, which is at least partially responsible for the increase in transmembrane delivery of polyplex and consequential enhancement in expression of transgene. Other cations, Mg^2+^ or Na^+^ did not show similar enhancement. Interestingly, addition of Ca^2+^ was rather detrimental for the transfection of lipoplex on Jurkat cells. Observation of significant enhancement in the transfection of non-viral vectors with a simple and physiologically relevant reagent like Ca^2+^ in the engineering of hard-to-transfect cells such as Jurkat warrants further investigation on similar strategies.

## Introduction

The delivery of exogenous nucleic acids into cells, i.e. transfection, is an important procedure in biology, medicine, and biotechnology, which offers great prospects in potential applications in the treatment of a variety of diseases including cancers, infectious diseases, and heritable genetic disorders. Strategies for transfection of mammalian cells can be categorized into viral (e.g. retroviruses), physical (e.g. electroporation), and chemical (e.g. polyplexes or lipoplexes) means. As each method of transfection possesses its own advantages and disadvantages (Kim & Eberwine, 2010), the selection of an appropriate method of transfection is necessary for individual application.

Compared to the methods in viral and physical categories, the chemical transfections have advantages such as 1) simple vector design and synthesis, 2) less or non-immunogenic than viral counterparts, 3) almost no limit in the size of exogenous gene to pack and deliver, and 4) no need for special instruments. While many new synthetic vectors have been developed recently (Lostalé-Seijo & Montenegro, 2018), the chemical transfections typically utilize cationic polymers or lipids to condense the nucleic acids of anionic nature, to form resulting complexes known as polyplexes or lipoplexes, respectively. The complexes of synthetic vectors and nucleic acids are taken up by the cells through various endocytic pathways. For successful transfection, the nucleic acid payloads need to be unpacked from the complexes and to escape from degradation within the endo-lysosomes. According to the so-called “proton sponge hypothesis” (Behr, 1997; Akinc et al., 2005), the buffering capability of polymeric vectors to absorb protons (to be protonated) within the acidic environments leads to an osmotic swelling and rupture of endosomes, which ultimately results in the release of payloads and vectors into the cytoplasm.

Polyethyleneimine (PEI) is one of the most studied cationic polymers for non-viral transfections. Effects of various parameters such as structure (branched vs. linear) and molecular weight of PEI, complexation ratio (PEI to nucleic acid ratio described as either w/w or N/P), dosage, and buffer conditions for polyplex formation have been extensively studied (Wightman et al., 2001; Wiseman et al., 2003; Intra & Salem, 2008; Dai et al., 2011; Sang et al., 2015). Arguably, 25 kDa branched PEI (bPEI) has been considered one of the gold standard non-viral vectors (Kichler et al., 2001; Raup et al., 2016). It is intriguing that the transfection efficiency achieved by PEI polyplexes is quite cell-type dependent; while they are effective in transfection of exogenous plasmid DNA (pDNA) into many cell types including 3T3 murine fibroblasts and COS-7 monkey kidney cell lines, the identical formulation is not very efficient in other cells such as chicken embryonic neurons (Boussif et al., 1995). Jurkat, an immortalized human T-cell line that was originally derived from a T-cell leukemia patient (Schneider et al., 1977), has been widely employed to study T-cell biology, especially as a model to examine T-cell signaling (Abraham & Weiss, 2004). Unfortunately, Jurkat cells are very difficult to transfect in general and PEI itself is not an effective vector for transfection of Jurkat cells (Olden et al., 2018).

Meanwhile, a lysosomotrophic agent, chloroquine, has long been used as an enhancer of efficiency in gene transfection of polycation-based formulations (Cotten et al., 1990; Erbacher et al., 1996; Wolfert & Seymour, 1998). More recently, pH-sensitive liposome made of dioleoylphosphatidylethanolamine (DOPE) and cholesteryl hemisuccinate (CHEMS), and a microtubule inhibitor, Tubastatin A, were successfully employed to enhance the efficiency of polyplex transfection for mesenchymal stem cells and primary neuronal cells (Ho et al., 2017). Similarly, calcium chloride has been used as a potent enhancer in a cationic-liposome mediated pDNA delivery in bovine hamster kidney cell line (Lam & Cullis, 2000). It was also reported that calcium can enhance gene expression when plasmid DNA (pDNA) is delivered using low molecular weight Poly-L-Lysine (PLL) or PEI (800 Da) in A549 human lung carcinoma cells (Xie et al., 2018). Here, we seek to evaluate Ca^2+^ as a potential reagent to enhance the non-viral transfection of Jurkat cells. In the current study, we report that Ca^2+^ enhances the pDNA transfection using polyplexes of pDNA with 25 kDa bPEI up to 12-fold in Jurkat cells compared to calcium-free control. We will also discuss about the unique and context-dependent functionality of Ca^2+^, by comparing to other cations as functional analogs and their effects on the lipoplex-based transfection on Jurkat cells.

## Materials and methods

### Materials

25 kDa branched PEI (Sigma-Aldrich), Lipofectamine 3000 (Invitrogen), calcium chloride (CaCl_2_), magnesium chloride (MgCl_2_) (EMD Millipore Corp), potassium chloride (KCl), dibasic sodium phosphate (Na_2_HPO_4_), potassium phosphate (KH_2_PO_4_), sodium chloride (NaCl), and 1 M HEPES solution (Fisher Scientific), 4% Paraformaldehyde (PFA) (ThermoFisher Scientific) were used as received from the manufacturers.

### Buffers

1X Phosphate buffered saline (137 mM NaCl, 2.7 mM KCl, 10 mM Na_2_HPO_4_, 1.8 mM KH_2_PO_4_, 1 mM CaCl_2_, and 0.5 mM MgCl_2_, pH 7.4) was prepared in ultra-pure water and heat-sterilized in Yamato SM510 autoclave. 1X HEPES buffered saline (30 mM HEPES, 150 mM NaCl, pH 7.5) was prepared in sterilized ultra-pure water and later filtered using a 0.45 μm syringe filter.

### Plasmids

pDNA expressing GFP (pAcGFP1-N3 plasmid, hereafter pGFP) was originally purchased from Takara Bio USA, Inc. pDNA expressing a *Gaussia* luciferase enzyme (pCMV-Gaussia-Dura Luc, hereafter pLuc) was purchased from Thermo Scientific. pGFP, pLuc, and pUC18 (Agilent Technologies, Inc.) were separately amplified by an overnight culture of transformed XL1 Blue competent cells (Agilent Technologies, Inc.) in LB media and subsequently purified using GeneJET Plasmid Maxiprep Kit (Thermo Scientific). The quality of the extracted plasmids was spectrophotometrically verified using the absorbance ratio between 260 and 280 nm. The A260/280 of the extracted plasmids were within 1.8 and 2.0. Extracted plasmids were also checked for purity in 1% (w/v) agarose gel (0.5X TAE, 75 V constant voltage) (Supplementary Figure 1(a)).

### Cell culture

Jurkat cell line was purchased from American Type Culture Collection (Manassas, VA). Cells were cultured in RPMI complete media without antibiotics supplemented with 10% Fetal Bovine Serum (FBS). Cells were cultured at 37 °C and 5% CO_2_ in Heracell VIOS 160i CO_2_ incubator. In every 3 days, cells were split to 0.5–1 × 10^6^ cells/ml in a T25 culture flask with fresh media. For regular maintenance, Jurkat cells were split before the density increased beyond 3 × 10^6^ cells/ml, and the cells grown to nearly 2.5 × 10^6^ cells/ml were used for transfection.

### Preparation of lipoplexes

All lipoplex formulations were prepared following the manufacturer’s recommended protocol. Briefly, we first prepared the two pre-solutions: 3.6 μl of Lipofectamine 3000 reagent mixed into 60 μl of Opti-MEM^TM^ by pipetting (pre-solution 1), and 4.8 μl of P3000 reagent along with 2,400 ng of pGFP mixed into 60 μl of Opti-MEM^TM^ (Fisher Scientific) by pipetting (pre-solution 2). Lipoplexes were formed by mixing pre-solution 1 and 2, followed by incubation for 15 min at room temperature before use.

### Preparation of polyplexes

For all transfection experiments, 2:1 (w:w) mixing ratio of PEI to pGFP was used for polyplex formation. For a typical polyplex formation, 2,400 ng of pGFP and 4,800 ng of PEI were mixed in pure water (total 80 μl) and incubated for 15 min at room temperature before use.

### SyBr exclusion assay

A mixture of 3,600 ng of pGFP and 7,200 ng of PEI in water (120 μl in total) was incubated for 15 min at room temperature to prepare the polyplex. Each 10 μl portion of this solution was mixed with 290 μl of calcium chloride solutions at different concentrations to make the indicated final concentrations. 3 μl of 100X solution of SyBr Safe (Invitrogen) was mixed with 300 μl of the polyplex solution. Triplicates for each condition (i.e. 3 wells of 100 ng pGFP per well) were prepared in a MicroFluor black 96-well plate (Thermo Scientific) and the fluorescence intensity was measured using BioTek Synergy HTX Multi-mode Reader equipped with 485/20 and 528/20 filters for excitation and emission, respectively.

### DNA labeling

pGFP was labeled with Cy5 at a targeted density of one dye molecule per 60 bps using Mirus Label-IT^R^ Nucleic acid labeling Kit following the manufacturer’s recommended protocol.

### DLS measurements

Polyplexes were prepared in pure water or the indicated buffers. The z-average size and zeta potential of polyplexes were measured using a Malvern Zetasizer Nano for 3 cycles per every measurement.

### Cell viability and transfection studies

Each formulation is typically made in bulk so as to take measurements at 4 different points in time, 3 replicates per time point (total 12 replicates per formulation). 800 μl of culture medium was supplemented with freshly prepared polyplexes (10 μl) or lipoplexes (120 μl), calcium chloride (stock conc. 5 M) or magnesium chloride (stock conc. 1 M) or sodium chloride (stock conc. 3 M) to make the indicated final concentrations, and finally a suspension of 1.2 × 10^5^ Jurkat cells in culture media was added to the mixture to make it up to 1.2 ml. This mixture was aliquoted into 12 wells (100 μl each) in a 96-well plate (Corning CoStar). Similar protocol was followed for all experiments with proportional scaling of components for smaller number of samples per formulation. The plates were incubated in a CO_2_ incubator (5%) at 37 °C until the number of live cells and/or the GFP expression were examined by flow cytometry at each indicated time point. Culture media was not replaced at any point of transfection experiment.

### Flow cytometry

At each measuring time point, the entire cells from each well were collected in FACS tubes and washed with FACS Buffer (1X PBS, 0.5% BSA, 2 mM EDTA) by centrifuging at 400 g for 5 min at 4 °C. After carefully discarding the supernatant, the tubes were blotted on Kimwipes^TM^ to remove the residual fluid, and the cells in each tube were resuspended in 300 μl of FACS buffer. 200 μl of each sample was analyzed by Attune NxT Flow Cytometer (Invitrogen).

### Confocal laser scanning microscopy

Cells retrieved from the indicated conditions were fixed by 2% PFA for 20 min at room temperature. For high-resolution imaging of GFP expression, the fixed cells were washed thrice with PBS and twice with Tris Buffered Saline (TBS); Cells were then permeabilized with 0.1% Triton-X 100 in TBS for 15 min with gentle shaking at room temperature; After three times washing using TBS, cells were blocked with 2% goat serum with 0.1% Tween-20 in TBS (TBST) for 30 min; Actin was stained using Alexa Fluor 594 Phalloidin (ThermoFisher Scientific) for 30 min in TBST; Cells were then washed thrice in TBS. For the examination of polyplex association with Jurkat cells, the fixed cells were washed twice with PBS. The cell pellets were prepared by centrifugation at 400 g for 5 min at 4 °C, re-suspended in a mixture of PBS and Prolong Diamond antifade with DAPI (Molecular Probes), mounted on a glass-slide, and covered by a coverslip. Slides were left in dark at room temperature for 24 h before they were examined under Zeiss LSM 700 confocal microscope, equipped with 405, 488, 555, and 639 nm lasers.

### Graphical representation and statistical analysis

Bar and line graphs were plotted, and all the statistical analyses were performed using v8.0 Prism software (GraphPad). For validation of the statistical significance in a multiple-group comparison, one-way or two-way ANOVA analysis with Dunnett’s multiple comparison were employed. Scatter plots and histograms were plotted using FlowJo software.

## Results

### Optimization of polyplex formulation

PEI is available in two structural forms, namely, linear PEI (lPEI) and branched PEI (bPEI). 25 kDa bPEI is reported to be superior to 25 kDa lPEI in the cell lines HeLa, HEK293, COS7, and HepG2 (Intra & Salem, 2008). Contrarily, 22 kDa lPEI has been shown to out-perform 25 kDa bPEI and 50 kDa bPEI in transfection experiments *in vitro* (16HBE cells) and *in vivo* when the reporter gene was delivered via mouse airways (Wiseman et al., 2003). It was also shown that the bPEI outperforms lPEI at a low N/P ratio of 3 and the difference is negligible at a higher N/P ratio of 10 (Dai et al., 2011). To keep our study design straightforward, we decided to use 25 kDa bPEI (hereafter PEI) for all experiments.

**Figure 1.**
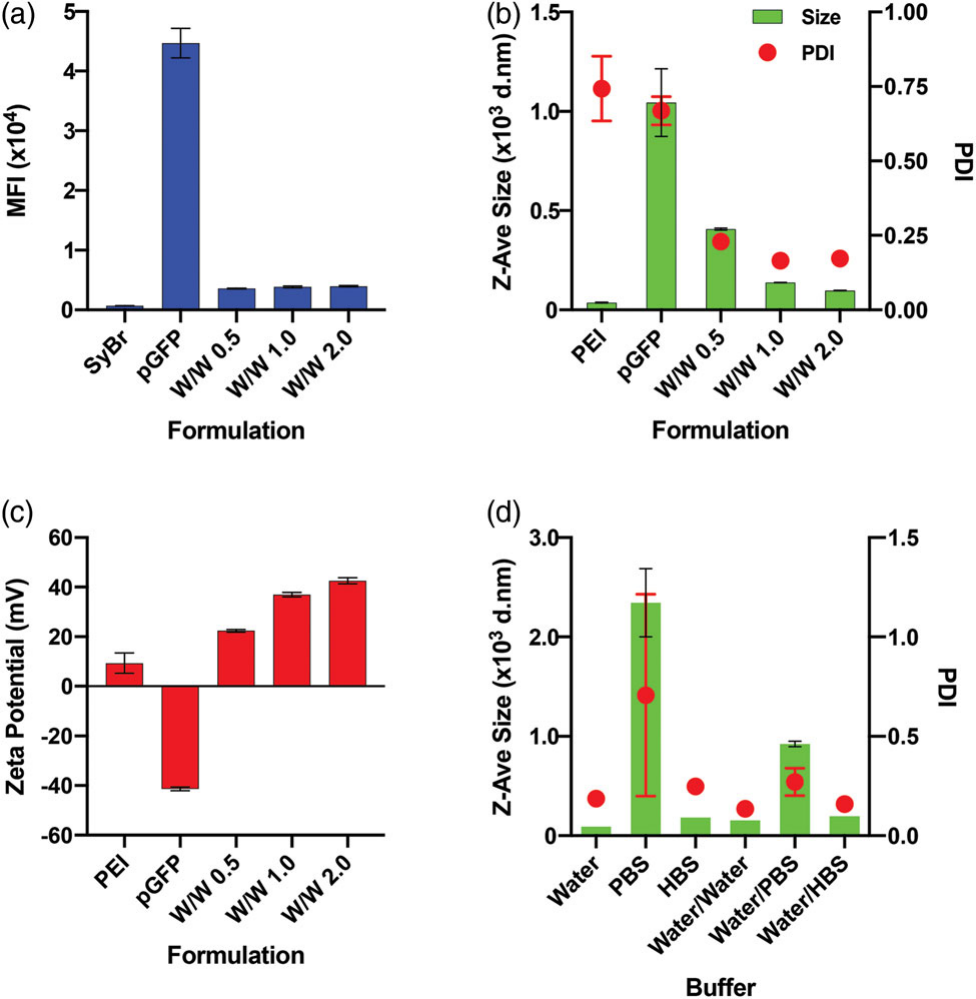
Optimization of PEI/pGFP polyplex formulation. (a-c) Degree of condensation (complexation) analyzed by SyBr Green dye exclusion assay (a), size (left y-axis) and polydispersity index (PDI, right y-axis) (b), and net surface charge (zeta potential) (c) of the polyplexes of PEI/pGFP at indicated weight ratio on each graph. (d) Size (left y-axis) and PDI (right y-axis) of polyplex of PEI/pGFP prepared at weight ratio of 2 within different environment. Polyplexes were either originally prepared in 1.3 ml of pure “Water”, “PBS”, or “HBS”, respectively, or first prepared in 100 μl of pure water and diluted (to final volume of 1.3 ml) using water or indicated buffers for “Water/Water”, “Water/PBS, or “Water/HBS” samples, respectively. Results are presented as mean ± SD (*n* = 3).

We first attempted to decide the optimal mixing ratio between the PEI and pGFP for stable polyplex formation. The SyBr exclusion assay revealed that a PEI/pGFP weight ratio (w/w) of 0.5 was enough to form a polyplex and that higher weight ratios may not be necessary (Figure 1(a)). This result agrees well with previous reports showing that nearly all pDNA can be complexed with PEI at N/P ratio of 3–4 (Erbacher et al., 1999; Ogris et al., 1999), as the weight ratio of 0.5 (PEI/pDNA) corresponds to 3.83 N/P ratio. However, an agarose gel electrophoresis analysis indicated that the polyplex made at w/w of 0.5 might not be stable, and w/w of 2 or higher is necessary for a tighter polyplex formation (Supplementary Figure 1(b)). In fact, the mean hydrodynamic diameters of polyplexes decreased as w/w ratio was increased from 0.5 (407.27 ± 5.2 nm) to 1 (138.27 ± 0.91 nm) to 2 (97.55 ± 1.21 nm), and the polydispersity index (PDI) of the polyplexes made at w/w of 0.5 (0.229 ± 0.02) were slightly higher than those made at w/w 1 (0.165 ± 0.01) or 2 (0.173 ± 0.01) (Figure 1(b)). Besides the size and compactness of polyplexes, the net charge of the polyplexes is another important factor that affects both transfection efficiency and cytotoxicity of polyplexes. It was previously shown that as PEI/pDNA ratio increases, more fraction of PEI exists free without participating in complexation, and that this free PEI fraction significantly enhances both toxicity and transfection efficiency (Boeckle et al., 2004; Yue et al., 2011). Expectedly, net charge of the polyplexes increased as w/w ratio increased from 0.5 (22.4 ± 0.53) to 1 (36.93 ± 0.87) to 2 (42.57 ± 1.21 mV) (Figure 1(c)). In our preliminary experiment, polyplex at w/w of 1 was ineffective in transfecting Jurkat cells, while polyplex at w/w of 2 showed at least some minimal efficiencies (Supplementary Figure 2). Altogether, we decided the polyplex of PEI/pGFP weight ratio (w/w) of 2 as the optimal formulation for our further investigation.

As polyplex formation is a result of charge-charge interactions between polycations (PEI) and polyanions (DNA), salt concentrations and pH values of the buffers that are used during the polyplex formation influence the physicochemical properties of the resulting polyplex (Sang et al., 2015; Raup et al., 2016). In order to test the sole effect of calcium during the actual transfection, we tried to employ a protocol to prepare polyplex formulations that are the most stable and resistant to changing of ionic strength of the environment. Previously, pure water or buffers such as PBS and HBS have been employed for polyplex formation to yield significantly varying sizes of the polyplex (Sang et al. 2015). We first prepared the polyplexes in 1.3 ml of pure water, PBS, or HBS to check how the difference in ionic strength of polyplex preparation condition (Supplementary Table 1) would alter the complexation of PEI and pDNA. The DLS measurements indicate that polyplexes made in pure water are the smallest in hydrodynamic diameter and the narrowest in PDI, while in PBS the largest and the broadest polyplexes were formed (Figure 1(d), Supplementary Table 2). In comparison, we also tested how the size of polyplex changes when the polyplexes are prepared in 100 μl of pure water, and then diluted (1:13) into pure water, PBS, or HBS. Even if all post-complexation dilutions slightly increased the size and PDI values, it was clear that the sizes of polyplexes prepared in pure water and diluted into PBS or HBS are smaller than the polyplexes prepared originally in PBS or HBS, respectively (Figure 1(d), Supplementary Table 2). In order to keep the polyplex formation as simple as possible without involvement of other metal ions, and to keep the size of the polyplex as minimal and stable as possible, we decided to prepare all polyplex formulations first in pure water and add it to the indicated conditions for transfection in the further studies.

Lastly, we also tried to find the optimum dosage of polyplex for the study. It is well known that increase in dosage of polyplex enhances transfection efficiency as well as cytotoxicity. To optimize the dosage for the Jurkat cells, we tested 3 different dosages – 20, 40, and 100 pg/cell of PEI complexed with 10, 20, and 50 pg/cell of pGFP, respectively. The growth curve of Jurkat cells for 4 days and the transfection efficiency measured by the size of GFP + cell population clearly demonstrated that both cytotoxicity and transfection efficiency are dose-dependent (Supplementary Figure 3). We selected the polyplex of 40 pg/cell PEI and 20 pg/cell pGFP as an optimal dosage for further investigations with Jurkat cells.

### Effect of calcium chloride on the stability, size and surface charge of PEI/pGFP polyplexes

**Figure 2.**
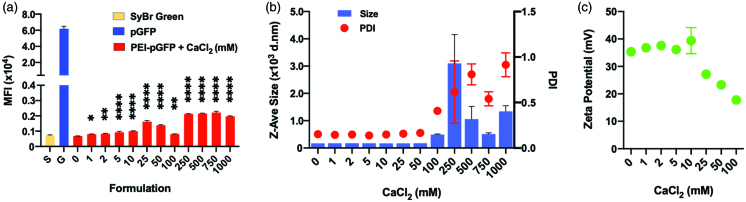
Effect of CaCl_2_ on the stability of PEI-pGFP polyplexes (w/w 2) analyzed by SyBr exclusion assay; size and net charge of polyplexes analyzed by dynamic light scattering zetasizer. Polyplexes are prepared in pure water. (a) All comparisons are made with “0” group. Results are presented as mean ± SD (*n* = 3; (a) 1-way ANOVA with Dunnett’s multiple comparisons, ** *p* = .0064, *** *p* =.0007, **** *p* <.0001, ** *p* = .0035).

We first evaluated the effect of calcium chloride on the stability of the polyplexes made in water. In Sybr green assay, the pDNA was increasingly accessible to intercalation by Sybr green dye as the concentration of calcium chloride increased (Figure 2(a)). This indicates a potential partial dissociation and looser complexation between the pDNA and PEI due to Ca^2+^ ions. In contrast, the sizes of the polyplexes measured by DLS were very uniform at around 175 nm (polydispersity index (PDI) ∼ 0.150) without being significantly affected by calcium chloride at concentrations up to 50 mM (Figure 2(b)). However, both the size and the PDI greatly fluctuated at concentrations of 100 mM and above (Figure 2(b)). The surface charge (zeta potential) of the polyplex remained at around 35 mV up to 10 mM calcium chloride, but they decrease steadily at concentrations of 25 mM and above (Figure 2(c)), which indicates increasing exposure of pDNA on the surface of the polyplexes. Altogether, while calcium chloride did not significantly deteriorate the stability of the polyplexes up to a quite high concentration (tested up to 1,000 mM), a tighter condensation of pDNA and PEI with monodisperse and small sizes is maintained only up to a smaller concentration threshold between 10 and 25 mM.

### Tolerance of Jurkat cells on varying concentrations of calcium chloride

**Figure 3.**
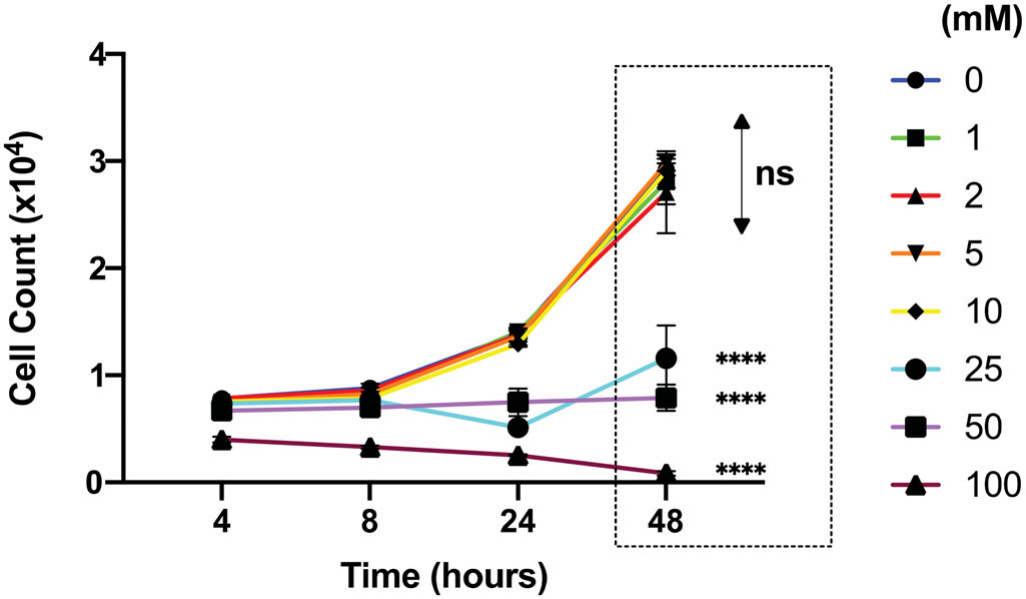
Effect of CaCl_2_ (mM) on Jurkat cell viability analyzed by flow cytometer. All comparisons (48 h) are made with “0” group. Results are presented as mean ± SD (*n* = 3; 2-way ANOVA with Dunnett’s multiple comparisons, **** *p* <.0001).

We examined the ability of Jurkat cells to withstand varying concentrations of calcium chloride for 2 days. The numbers of live Jurkat cells increased over time without any significant difference at all time points measured for CaCl_2_ concentrations up to 10 mM (Figure 3). However, there was no growth in cell number in 25 mM and 50 mM conditions, while cell number started to decrease even at the earliest tested time point (4 h post-plating) at 100 mM. In fact, all Jurkat cells with calcium chloride concentrations above 250 mM were dead by 8 h post-plating. Therefore, we decided to limit our further experiments with calcium chloride at concentrations only up to 25 mM.

### Effect of calcium chloride on pGFP transfection in Jurkat Cells

**Figure 4.**
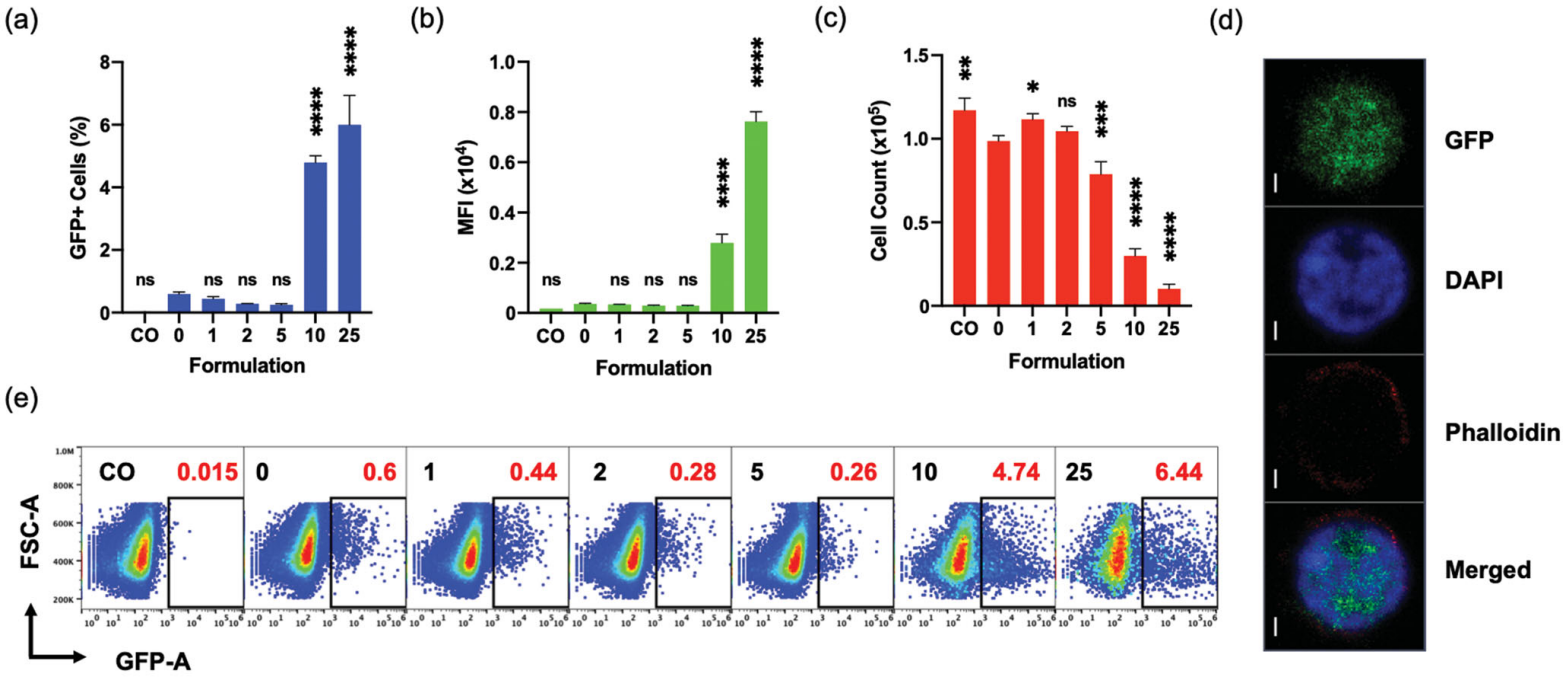
Effect of CaCl_2_ on the transfection of Jurkat cells analyzed by flow cytometer at 96 hpt. (a, b, c, e) “CO” is a cell only control; “0”, “1”, “2”, “5”, “10”, and “25” are polyplex formulations with 0-, 1-, 2-, 5-, 10-, and 25 mM CaCl_2_, respectively. (d) Split view of a GFP expressing Jurkat cell from “10” captured with an oil immersion lens at 100X using Confocal Microscope LSM700; Scale bar is 1 μm. (e) Sample code and respective transfection efficiencies (red) are indicated in each square box; one of the three replicates whose transfection efficiency is closer to the mean is chosen for the plot. (a, b, c) All comparisons are made with “0”; Results are presented as mean ± SD (*n* = 3; 1-way ANOVA with Dunnett’s multiple comparisons, * *p* = .0258, *** *p* =.0009, **** *p* <.0001).

We proceeded to evaluate the effects of calcium chloride on the transfection efficiency of PEI-based polyplexes on Jurkat cells. Compared to the cell only (CO) group, PEI polyplex without any calcium chloride (0 mM) induced GFP expression in a very small population around 0.6% (Figure 4(a,e)). It was intriguing to observe the Jurkat cell populations, which displayed a small positive shift in the fluorescence signals of GFP channel when they are treated with polyplexes, and the size of the low-level fluorescence shift further increased with an addition of calcium (Supplementary Figure 4(a)). However, this increase in low-level fluorescence was invariably observed when an empty bacterial expression plasmid vector, pUC18, was employed in place of pGFP (Supplementary Figure 4(b)). Meanwhile, addition of calcium itself without polyplexes did not induce any shift of signals in GFP channel from Jurkat cells (Supplementary Figure 4(c)). Thus, we concluded that this low-level signal does not represent the expression of GFP. Therefore, in order to precisely quantify the GFP expression, we decided to employ the Jurkat cells treated with pUC18 polyplex plus 10 mM calcium for proper gating control, or a similarly stringent gating strategy in the further analysis of flow cytometry.

Under this gating strategy, the percentage of GFP + cells slightly decreased at 1, 2 and 5 mM, but significantly increased at 10- and 25-mM calcium concentrations, compared to the 0 mM (no additional calcium) control, when the Jurkat cells were examined at 96 h after transfection (Figure 4(a,e)). A wide spectrum of GFP expression level appears in 10- and 25-mM groups only (Figure 4(e)), which was verified by the statistically significant increases in the mean fluorescence intensity (MFI) values of these groups (Figure 4(b)). The GFP expression in the Jurkat cells was examined by confocal laser scanning microscope (CLSM) imaging at a high resolution (Figure 4(d)). It is noteworthy that the numbers of live cells in 10 mM and 25 mM groups were significanly lower than 0 mM, presumably due to a combined cytotoxicity of calcium and polyplex (Figure 4(c)). Nevertheless, it is clearly demonstrated that the addition of calcium chloride at concentrations of 10 mM or above can enhance the transfection efficiency of pDNA using PEI-based polyplexes on otherwise hard-to-transfect Jurkat T cells.

### Influence of other ions in transfection of Jurkat cells using polyplexes

We tested other metal ions that might be able to act as functional analogs of calcium ions. For this, we performed the same transfection assays in the presence of magnesium chloride or sodium chloride in place of calcium chloride. To match the ionic density, we used the same concentrations of magnesium chloride to the conditions of calcium chloride but double the concentrations of sodium chloride.

**Figure 5.**
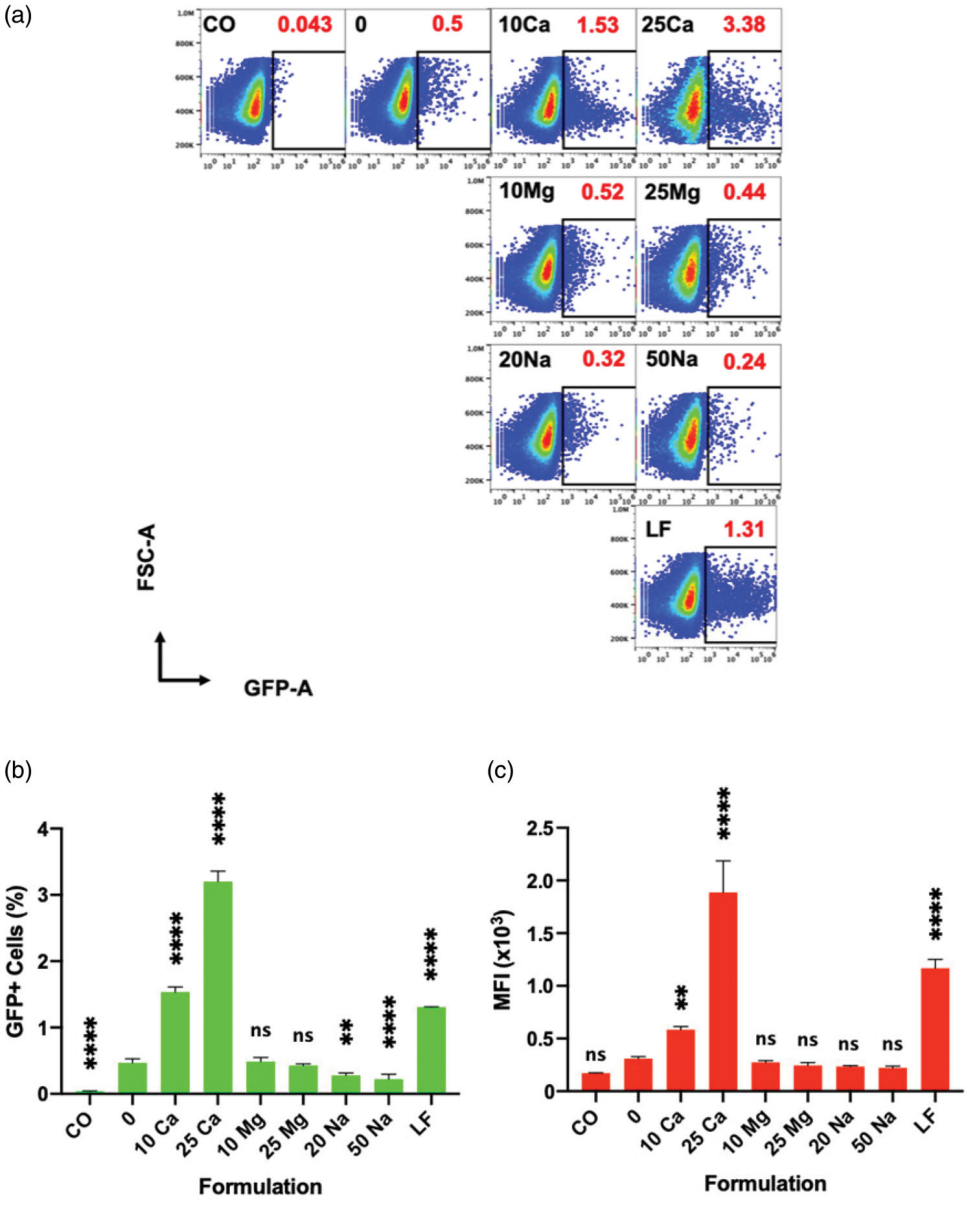
Effect of various cations on transfection of polyplex on Jurkat cells, and their direct comparison to lipoplex analyzed by flow cytometer at 96 h. “CO” is a cell only control; “0” is a polyplex only control; “10 Ca” and “25 Ca” are polyplex formulations with 10- and 25 mM CaCl_2_, respectively; “10 Mg” and “25 Mg” are polyplex formulations with 10- and 25 mM MgCl_2_, respectively; “20 Na” and “50 Na” are polyplex formulations with 20- and 50 mM NaCl, respectively; and “LF” is a lipoplex condition. (a) Sample code and respective transfection efficiencies (red) are indicated in each square box; one of the three replicates whose transfection efficiency is closer to the mean is chosen for the plot. (b, c) All comparisons are made with “0” group; Results are presented as mean ± SD (*n* = 3; 1-way ANOVA with Dunnett’s multiple comparisons, ** *p* =.0017 to 0.005, **** *p* <.0001).

In direct comparisons, magnesium chloride groups did not show any effect in transfection compared to polyplex-only control (0 mM), while addition of sodium chloride at indicated concentrations made the size of GFP+ population rather decreased (Figure 5(a,b)). Unlike the wide spectrum of GFP expression levels observed in the calcium chloride groups, most of the GFP + cells in the magnesium chloride groups resemble the low-level GFP expression of 0 mM control (Figure 5(a,c)). Altogether, calcium cation instead of chloride anion seems to be responsible for the enhancement in polyplex transfection efficiency, and that the other tested cations, Mg^2+^ and Na^+^, were ineffective.

### Comparison between polyplex and commercial lipoplex

We made a comparison between the calcium-assisted polyplex and a commercially available state-of-the-art reagent for transfection of pDNA in Jurkat cells. We chose Lipofectamine 3000 (LF) which is a positively charged liposome-based transfection reagent. Thus, the complex of LF with pDNA is called a lipoplex. In a transfection assay up to 96 h, the size of the GFP+ population of LF group was significantly higher than the polyplex without any calcium (0 mM) (Figure 5(a,b)). However, polyplexes perform better in terms of percentage of GFP+ cells when calcium was added at 10 mM or 25 mM concentrations. In terms of MFI values, LF group was superior to polyplex with 10 mM calcium (Figure 5(c)), due to the difference in levels of GFP expression among GFP+ cells (Figure 5(a)).

We further tested the effects of CaCl_2_ on the LF-mediated transfection in Jurkat cells. Interestingly, calcium didn’t improve the transfection efficiency but rather showed a certain detrimental effect both in terms of GFP+ cell % and MFI (Supplementary Figure 5(a,b)). This stark difference in transfection behaviors between calcium-assisted polyplex and lipoplex might originate from the fundamental difference in mechanism of action between polyplex and lipoplex (Cohen et al., 2009; Rehman et al., 2013), which warrants further investigation.

### Effect of calcium chloride on the association of polyplex or lipoplex and Jurkat cells

**Figure 6.**
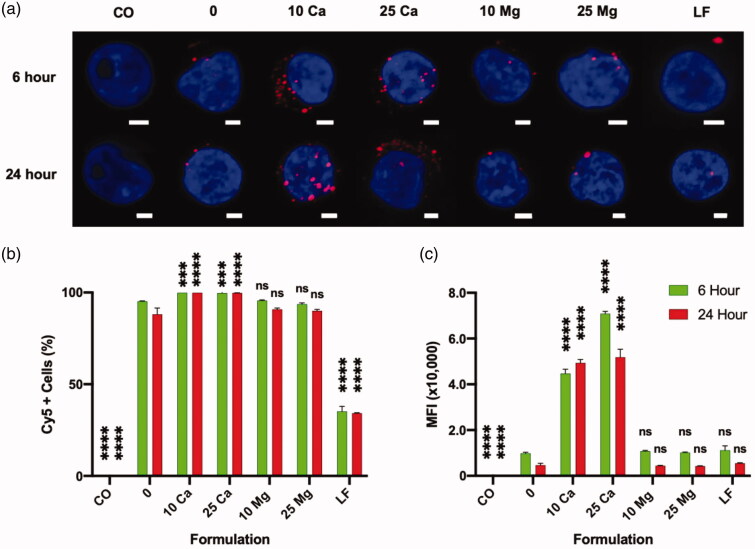
Effect of CaCl_2_ on the association of pGFP-Cy5 polyplexes and Jurkat cells in a 48 h culture analyzed by flow cytometer. “CO” is a cell only control; “0” is a polyplex only control; “10 Ca” and “25 Ca” are polyplex formulations with 10- and 25 mM CaCl_2_, respectively; “10 Mg” and “25 Mg” are polyplex formulations with 10- and 25 mM MgCl_2_, respectively; and ”LF” is a lipoplex only condition. (a) Confocal laser scanning microscope maximum intensity projection image of Jurkat cells associated with polyplexes; Scale bar in every image is 2 μm; Nucleus is stained with DAPI (blue) and pGFP is labeled with Cy5 (red). (b, c) All comparisons are made with “0” group of respective time point; Results are presented as mean ± SD (*n* = 3; 1-way ANOVA with Dunnett’s multiple comparisons, *** *p* = .0005 and 0.0009, **** *p* <.0001).

In order to understand the role of calcium in the enhancement of transfection, we examined the association of polyplex and Jurkat cells using Cy5-labeled pGFP. At 6- and 24 h post-plating, the Jurkat cells were first examined under CLSM with a maximum intensity projection (MIP) view (Figure 6(a)). It is clear that more pDNA polyplexes are associated with the Jurkat cells in the presence of calcium as early as 6 h after plating, compared to the polyplex-only control. Such enhancement was not observed from magnesium groups. Quantitative analysis using flow cytometry also clearly demonstrated that calcium but not magnesium enhances the association of polyplexes with Jurkat cells in terms of both the size of the polyplex-associated cell population (Cy5+ cells, Figure 6(b)) and the amount of polyplexes per Jurkat cells (MFI, Figure 6(c)). In particular, the multi-fold increase in the MFI of the calcium-assisted groups indicates that this enhanced association might have induced the transmembrane delivery of significantly higher copy numbers of pDNA through endocytosis.

In a direct comparison between polyplex and lipoplex, LF group showed similar MFI as the polyplex without any calcium (0 mM control) (Figure 6(b)), while only about a third of size of polyplex-associated population was associated with lipoplex. This suggests that the copy number of pDNA per lipoplex could be significantly greater than the copy number of pDNA per polyplex.

More intriguingly, in stark contrast to the polyplex, an addition of calcium rather reduced the association between lipoplex and Jurkat cells (Supplementary Figure 5(c)). This at least partially explains the detrimental effect of calcium on the lipoplex-mediated transfection on Jurkat cells (Supplementary Figure 5(a,b)).

Altogether, calcium played a significant role in association of non-viral vectors on Jurkat cells, differently for polyplex and lipoplex: calcium enhances the association of polyplexes but reduces the association of lipoplexes. These effects of calcium on association of either of non-viral vectors would have direct impacts on the copy numbers of delivered pDNA and the resulting transfection efficiency. It is likely that there is a significant difference in the mechanism of association and uptake of polyplex and lipoplex onto Jurkat cells.

## Discussion

Polyplexes are formed by complexation between positively charged polymers (e.g. PEI) and negatively charged nucleic acids (e.g. pDNA) via electrostatic interactions. For successful transfection, the pDNA containing polyplexes should have a net positive charge to electrostatically interact with the negatively charged cell membrane (Raup et al., 2016). Hence, a surplus of polycation is preferable for transfection. As the free polycation is also responsible for the increased cytotoxicity, finding an optimal ratio of polycation to pDNA is necessary. In this report, we found weight ratio of 2 (N/P ratio around 15) as such an optimum.

Various endocytic pathways, such as clathrin-coated pits, caveolae-mediated, micropinocytosis, or combination of these are known to be involved in the internalization of polyplexes (Rejman et al., 2005; Rehman et al., 2012; Reilly et al., 2012; Ingle et al., 2013). At least some of these pathways are reported to display an upper particle size limit of approximately 200 nm for effective internalization (Pandey & Sawant, 2016). Thus, keeping the size of polyplex small and monodisperse during the transfection may be beneficial for an efficient transfection. It is known that the hydrodynamic size of polyplexes increases in higher ion concentrations (Sang et al. 2015), and it was evident that the polyplexes made in PBS were significantly larger than those made in pure water (Figure 1(d), Supplementary Table 2). Once the polyplexes were prepared in pure water, however, the complexes gained a certain level of resistance to change in ionic concentrations (Figure 1(d), Supplementary Table 2). So we first prepared the polyplex in pure water, and then added the formulation to the cell culture medium, similar to others (Rejman et al., 2005; Ingle et al., 2020). It is noteworthy that our study is different from other previous efforts to evaluate the effects of salt concentrations during the polyplex formation on the final physicochemical and biological properties of the polyplex (Sang et al., 2015; Raup et al., 2016). Here we attempted to normalize all formulation parameters by employing a uniform dosage and a uniform formulation made at a single defined preparation condition, so that the sole effect of extra calcium ions present during the transfection (i.e. the actual interaction between the polyplex and Jurkat cells) could be studied.

Prior to assaying transfections, we first investigated the concentration-dependent effects of calcium chloride on the physiochemical properties of such pDNA-PEI polyplexes. While the hydrodynamic sizes measured by dynamic light scattering did not change significantly up to 50 mM, the complex between PEI and pDNA seems to be significantly loosened above around 10 mM, which was revealed by increase in Sybr-dye intercalation into pDNA and decrease in surface zeta-potential. Interestingly, the tolerance threshold in calcium chloride concentration of Jurkat cells coincided at around 10 mM, i.e. the calcium chloride starts to negatively affect the viability/expansion of Jurkat cells at 25 mM or above. Therefore, we could investigate the effect of calcium chloride on transfection efficiency up to 25 mM, without significant cytotoxicity originating from the calcium chloride.

In general, the transfection process could be divided into several consecutive steps, in each of which, there are unique and critical biological barriers to overcome for a successful gene transfection. i) The polyplex needs to bind to the plasma membrane to be internalized by the cells (endocytosis); ii) the polyplex needs to escape from the endo-lysosome before it is degraded; iii) the payload nucleic acids (pDNA) needs to be disassembled from the polyplex; and iv) the pDNA needs to be translocated into the nucleus. Steps ii) and iii) could potentially occur simultaneously. Nevertheless, in this framework, calcium could participate in one or more of these steps to enhance the transfection of polyplex.

In order to study the roles of calcium in more detail, we investigated the effect of calcium on the association and potential endocytosis of polyplexes. The flow data showing the association of dye-labeled pDNA and Jurkat cells demonstrated that calcium enhances the interaction significantly at 10 mM and 25 mM concentration. How exactly positively charged divalent ions can contribute to the association between positively charged polyplexes and negatively charged plasma membrane is unclear. It is worth noting that a significant fraction of Jurkat cells associate with the polyplex even without calcium (over 75%) but only 0.403 ± 0.05% (mean ± SD, from 3 experiments) cells eventually show GFP expression mostly in a low level. Similarly, although nearly all the cells are associating with polyplexes at 10- and 25-mM calcium concentrations, only about 3.36 ± 0.43% and 4.81 ± 0.52% of them, respectively, expressed the transgene (GFP). Thus, it is likely that a very small fraction of the many polyplexes that initially associate with the Jurkat cells would overcome all the above-mentioned barriers to contribute to the gene expression. Nevertheless, the quantity of associated (internalized and membrane-bound) polyplexes (MFI in pDNA label, Cy5) was significantly enhanced in the presence of extra calcium (Figure 6(a,c)) and there is a direct correlation to the gene expression (both GFP+ % and MFI in GFP channel). A carefully executed study previously showed that the polyplexes bind to the cell membrane within a relatively short period (10 min–7 h) and its internalization follows over a longer period (up to 30 h) (Delafosse et al., 2016), and these two steps are inevitably related. Even if we don’t know exactly at which step calcium plays a role, at least it is clear that calcium enhanced the overall association between the polyplex and Jurkat cells, which consequentially improved the transgene expression.

Unfortunately, the combined cytotoxicity of calcium and PEI-based polyplex (Figure 4(c)) makes it difficult to apply the current method to real applications. Further optimization in dosage of calcium and polyplex as well as exchange of polyplex formulation with less toxic counterparts would be an imminently feasible next step.

The comparison between the polyplex and the lipoplex provides very interesting insights. In our direct comparison, the commercial state-of-the-art lipoplex formulation (LF) showed a similar transfection efficiency to the polyplex group assisted with 10 mM calcium (Figure 5). But in terms of pDNA delivery, LF group was inferior to polyplex groups assisted with 10 mM calcium (Figure 6(b,c)). This result agrees well with the previous findings about the difference between polyplex and lipoplex. First, it was demonstrated that the lipoplex-delivered pDNAs are more efficiently expressed than the polyplex-delivered counterparts due to the better unpacking (dissociation) of pDNA from lipoplexes than from polyplexes (Cohen et al., 2009). Additionally, when similar numbers of lipoplexes and polyplexes are delivered to a cell, much more number of lipoplexes can deliver nucleic acid payloads out of endosomes than polyplexes due to the difference in the mechanism and the kinetics of endosomal escape (Rehman et al., 2013). Furthermore, in our current study, addition of calcium enhanced the association of only the polyplex on Jurkat cells and the transfection of transgene, but not the lipoplex (LF). In fact, calcium showed a negative impact on the performance of LF. Altogether, the results presented in the current study suggests further studies on the difference in the mechanism of action between polyplex and lipoplex employed for transfection of various cell types.

The functional analog assay confirmed that calcium has a unique ability to enhance transfection efficiency of polyplexes through enhancement of association between the polyplexes and Jurkat cells, which is not shared by other tested cations, sodium or magnesium. This tentatively suggests that there may not be a universally applicable common critical feature for enhancement of transfection among different cations.

It is worth discussing that calcium has a unique potential to contribute to the expression of transgene by enhancing other physiological processes. For example, it is known that endoplasmic reticulum (ER), a landmark for protein biosynthesis in eukaryotes, functions as a calcium storing organelle by trapping most of the calcium molecules on ER-surface proteins (Carreras-Sureda et al., 2018). ER-localized chaperones such as calnexin and calreticulin bind to calcium with low affinity for their activity (Ashby & Tepikin, 2001; Carreras-Sureda et al., 2018). Thus, it is possible that calcium enhances the expression of exogenous gene (GFP) in Jurkat cells by elevating the function of ER. Additionally, in many cell types including T cells and B cells, a depletion of calcium in the ER leads to an initiation of calcium influx from the exterior of cells, a process known as ‘Store-Operated Calcium Entry’ (SOCE) (Marshall et al., 2015) whose potential in enhanced endocytosis has not been fully explored yet. Therefore, further investigations are warranted to precisely delineate the functional features of calcium in transfection of transgenes and to possibly utilize this strategy as a universal transfection booster for non-viral vectors.

## Conclusion

Here we report that the pDNA transfection efficiency of PEI-based nanocarrier, polyplex, can be enhanced by the calcium ion, a physiologically relevant divalent cation. Despite the fact that PEI has been widely adopted as one of the main cationic polymers as a non-viral gene delivery vehicle, the transfection efficiency of PEI-based polyplex by itself is not high enough to be utilized in transfection of certain cell types such as Jurkat that is intrinsically resistant for transfection. Therefore, it is very encouraging to observe that the transfection of Jurkat cells using PEI polyplex was effectively enhanced by the simple addition of calcium into the transfection medium. With further investigation, especially to lessen the cytotoxicity while enhancing the efficiency of the currently presented formula, the findings of the current study will potentially be applicable for engineering of primary human T lymphocytes with exogenous genes such as chimeric antigen receptors (CARs) for cancer immunotherapy. Nevertheless, further investigations on calcium’s role as a potential transfection booster in non-viral gene deliveries is warranted.

## Supplementary Material

Supplemental Material
