# Supplementary material for: Calcium enhances polyplex-mediated transfection efficiency of plasmid DNA in Jurkat cells
Source: Drug Deliv. 2020 Jun 3;27(1):805–15. doi: 10.1080/10717544.2020.1770371 (PMC8216448; doi:10.1080/10717544.2020.1770371)
Supplement: Supplemental Material [file IDRD_A_1770371_SM9439.docx]

**Supporting Information**

**Calcium enhances polyplex-mediated transfection efficiency of plasmid DNA in Jurkat cells**

V. S. S. Abhinav Ayyadevara^1^, Kyung-Ho Roh^1, 2, *^

1: Biotechnology Science and Engineering, University of Alabama in Huntsville, Huntsville, AL, 35899, USA.

2: Department of Chemical and Materials Engineering, University of Alabama in Huntsville, Huntsville, AL, 35899, USA.

* To whom correspondence should be addressed:

Kyung-Ho Roh, PhD

Assistant Professor

Department of Chemical and Materials Engineering

University of Alabama in Huntsville

301 Sparkman Drive NW

Huntsville, AL 35899

Telephone: 256-824-5292

Email: [kyung-ho.roh@uah.edu](mailto:kyung-ho.roh@uah.edu)

**Supplementary Figures and Tables**

**Supplementary Figure 1:** Agarose gel electrophoresis. (a) Quality of extracted plasmids – pLuc (5,482 bp), pGFP (4,722 bp), and pUC18 (2,686 bp); (b) Gel retardation assay of polyplexes of PEI and pLuc complexed at indicated weight ratio of PEI/pLuc on top of each lane.

**Supplementary Figure 2:** Comparison between polyplexes of PEI/pGFP made at weight ratios of 1 and 2 in transfecting Jurkat cell analyzed by flow cytometer at 24 hour. Cell count (left x-axis) and transfection efficiency measured by GFP+ cell %. Results are represented as mean ± SD (n=3).

**Supplementary Figure 3:** Effect of polyplex dosage on growth and transfection of Jurkat cells analyzed by flow cytometer. (a) Jurkat cell growth kinetics, (b) Cell count (left y-axis) and transfection efficiency (right y-axis) at 96 hour**.** Numbers in the legend (a) and x-axis (b) indicate the respective dosage in pg per cell of PEI/pGFP in polyplex form. Results are presented as mean ± SD (n=3).

**Supplementary Figure 4:** Enhancement in low-level GFP fluorescence analyzed by flow cytometry. (a) Effect of the combination of calcium and pGFP polyplexes on low-level GFP signal at 96 hpt. (b) Effect of an empty bacterial expression vector at 96 hpt. (c) Effect of calcium chloride in polyplex-free culture media at 48-hours post plating.

**Supplementary Figure 5:** Effect of CaCl_2_ on transfection using Lipofectamine 3000 analyzed by flow cytometer at 96 hpt. “0”, “1”, “2”, “5”, “10”, and “25” are lipoplex formulations with final concentrations of 0-, 1-, 2-, 5-, 10-, and 25- mM CaCl_2_, respectively. (a, b) Unlabeled pGFP was used to measure GFP expression. (c) Cy5 labelled pGFP was used only to measure the number of Cy5 stained cells. All comparisons are made with “0”. Results are presented as mean ± SD (n=3; 2-way ANOVA with Dunnett’s multiple comparisons, * *p* = 0.0105 ** *p* = 0.0014 and 0.0052, *** *p* = .0004, **** *p* < .0001).

**Supplementary Table 1:**

Ionic strength of various solutions and buffers used. The ionic strength was calculated using Debye-Huckel formula.


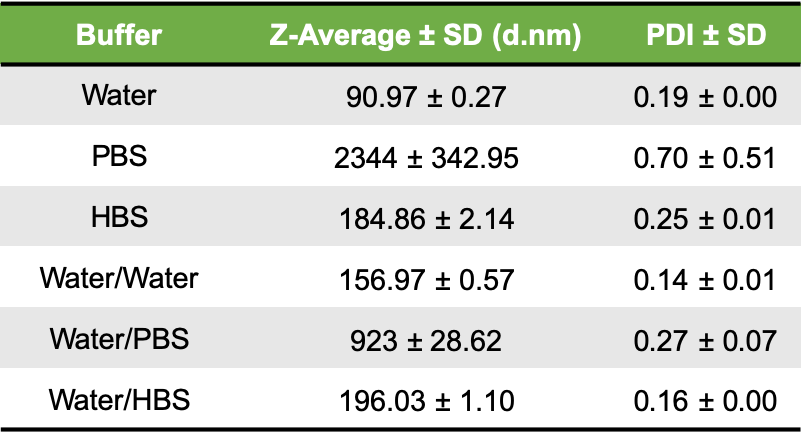


**Supplementary Table 2:**

Size and PDI values of polyplexes (PEI/pDNA weight ratio of 2) shown in Figure 1d. Polyplexes were made either directly in different medium (1.3 ml of Water, PBS, or HBS) or in 100 μl pure water first and diluted into different medium (final volume 1.3 ml).
